# Root-applied brassinosteroid and salicylic acid enhance thermotolerance and fruit quality in heat-stressed ‘Kyoho’ grapevines

**DOI:** 10.3389/fpls.2025.1563270

**Published:** 2025-04-03

**Authors:** Yanli Sun, Sijie Sun, Muhammad Salman Zahid, Qian Qiu, Lei Wang, Shiping Wang

**Affiliations:** ^1^ Department of Plant Science, School of Agriculture and Biology, Shanghai Jiao Tong University, Shanghai, China; ^2^ Microbial Ecology and Truffle Innovation, Mycorrhizal Systems Ltd., Lancashire, United Kingdom

**Keywords:** grape, photosynthesis, heat stress, hormones, fruit quality

## Abstract

**Introduction:**

The increasingly severe global greenhouse effect has become an irreversible trend, significantly impacting viticulture regions through heat stress during various grape growth stages, especially under protected cultivation conditions where high temperatures frequently occur. Therefore, studying the impact of heat stress on grapevine growth and fruit quality across the entire growth and development period, along with effective mitigation measures, is crucial.

**Methods:**

In this study, three-year-old 'Kyoho' grapevines were used as experimental materials, with four treatment groups: a control group, a hightemperature group (heat stress, HT), a high-temperature + brassinolide group (BR), and a high-temperature + salicylic acid group (SA). During the flowering, young berry swelling, and veraison stages, BR and SA were applied via nutrient solutions every seven days.

**Results:**

The results demonstrated that BR restored the maximum photosynthetic rate (Amax) to 96.14% of CK by the 18th day of flowering, significantly outperforming SA's recovery rate of 86.64%. Both treatments maintained light saturation points (1200 μmol•m^⁻²^•s^⁻¹^) and CO2 saturation thresholds equivalent to CK. The decline in PSII photochemical efficiency (Fv/Fm) was reduced from 18% in HT to 5–8% in BR/SA-treated groups, with BR showing minimal deviation (2.3%) from CK during veraison, effectively mitigating PSII photoinhibition caused by heat stress. Furthermore, both treatments reduced leaf malondialdehyde (MDA) content, minimizing membrane lipid peroxidation, while increasing soluble protein (SP) content to protect leaves. Under heat stress, BR notably improved the fruit set rate by 22.67% compared to HT (SA: 13%), promoted berry expansion, and enhanced the accumulation of sugars and anthocyanins in the fruit skin, with SA showing similar, though slightly less pronounced, effects.

**Discussion:**

These findings provide valuable theoretical insights into the use of exogenous hormones in root nutrient solutions as a strategy to mitigate the adverse effects of heat stress in grape production.

## Introduction

Plant growth and geographic distribution are heavily influenced by environmental conditions and are particularly vulnerable to environmental changes. Among the complex and dynamic environmental factors, high temperature is a critical cue that affects all aspects of plant development. Prolonged exposure to high temperatures can cause severe and often irreversible damage to plant growth, development, and productivity by disrupting various biological functions and reducing biomass and yield (Li N et al., 2021; Savada et al., 2017). Photosynthesis, one of the most sensitive physiological processes, is profoundly affected by heat stress. Elevated temperatures impair photosynthesis by reducing the photosynthetic rate, causing permanent damage to photosynthetic capacity, and disrupting associated metabolic processes that convert light energy into chemical energy (Demmig-Adams et al., 2018). Chloroplasts, the organelles responsible for photosynthesis, are particularly vulnerable under heat stress. High temperatures negatively affect chloroplast biogenesis, impair biosynthesis of chlorophyll (Chl) in plastids, and accelerate Chl degradation, ultimately leading to reduced Chl accumulation (Reda and Mandoura, 2011; Antoniou et al., 2017). Heat stress also damages the thylakoid membrane, disrupting membrane-associated electron carriers and enzymes, including those in photosystem II (PSII) and the water-oxidizing complex (WOC), as well as the structural and functional integrity of the light-harvesting complex (LHC) (Lipova et al., 2010).

Phytohormones, as endogenous signaling molecules, play crucial roles in regulating plant growth, development, and stress responses (Verma et al., 2016; Kumar et al., 2019; Emenecker and Strader, 2020). Exogenous application of phytohormones has been shown to mitigate heat-induced damage and enhance heat tolerance, underscoring their active involvement in stress response pathways (Li N et al., 2021). Among these phytohormones, Brassinosteroids (BRs) are polyhydroxylated steroidal hormones that promote cell elongation, participate in various physiological processes, and enhance plant tolerance to abiotic stresses, including heat stress, by regulating adaptive morphological changes (Miao et al., 2024). Salicylic acid (SA), a well-known phenolic compound, regulates physiological and metabolic functions, improves stomatal conductance, enhances photosynthesis, and promotes ion uptake and transport, ultimately supporting plant growth under stress (Jahan et al., 2019).

Grapes are cultivated globally for their economic and nutritional value, but grapevine productivity is highly sensitive to climate change, with extreme temperatures being a major limiting factor (Alikadic et al., 2019; Venios et al., 2020). As global temperatures continue to rise, developing effective strategies to mitigate heat stress damage in grapevines has become increasingly urgent. While previous studies have explored the use of plant growth regulators in viticulture, the potential of exogenous hormones to enhance heat tolerance in grapes remains underexplored.

In this study, we focused on the ‘Kyoho’ grape variety, which is widely cultivated in regions prone to high temperatures, making it an ideal model for investigating heat stress mitigation strategies. This makes it an ideal model for investigating strategies to mitigate heat stress. We investigated the effects of high-temperature stress on the growth and development of the ‘Kyoho’ grape variety during key phenological stages and assessed the potential of exogenous applications of BRs and SA to improve heat tolerance. We quantified the temporal impacts of heat stress on the photosynthetic efficiency, membrane stability, and fruit development of ‘Kyoho’ grapes, compared the differential protective effects of BR and SA on floral organs. By evaluating the efficacy of BR and SA across the entire growth cycle, our research provides a comprehensive comparative analysis of their potential to improve heat tolerance in ‘Kyoho’ grapes. We also establish quantitative links between physiological recovery and fruit quality parameters and offer valuable insights into using exogenous hormones via irrigation nutrient solutions to enhance grapevine resilience to heat stress. These findings provide a robust scientific basis for sustainable viticulture in the face of climate change.

## Materials and methods

### Plant materials and treatments

**Figure 1:**
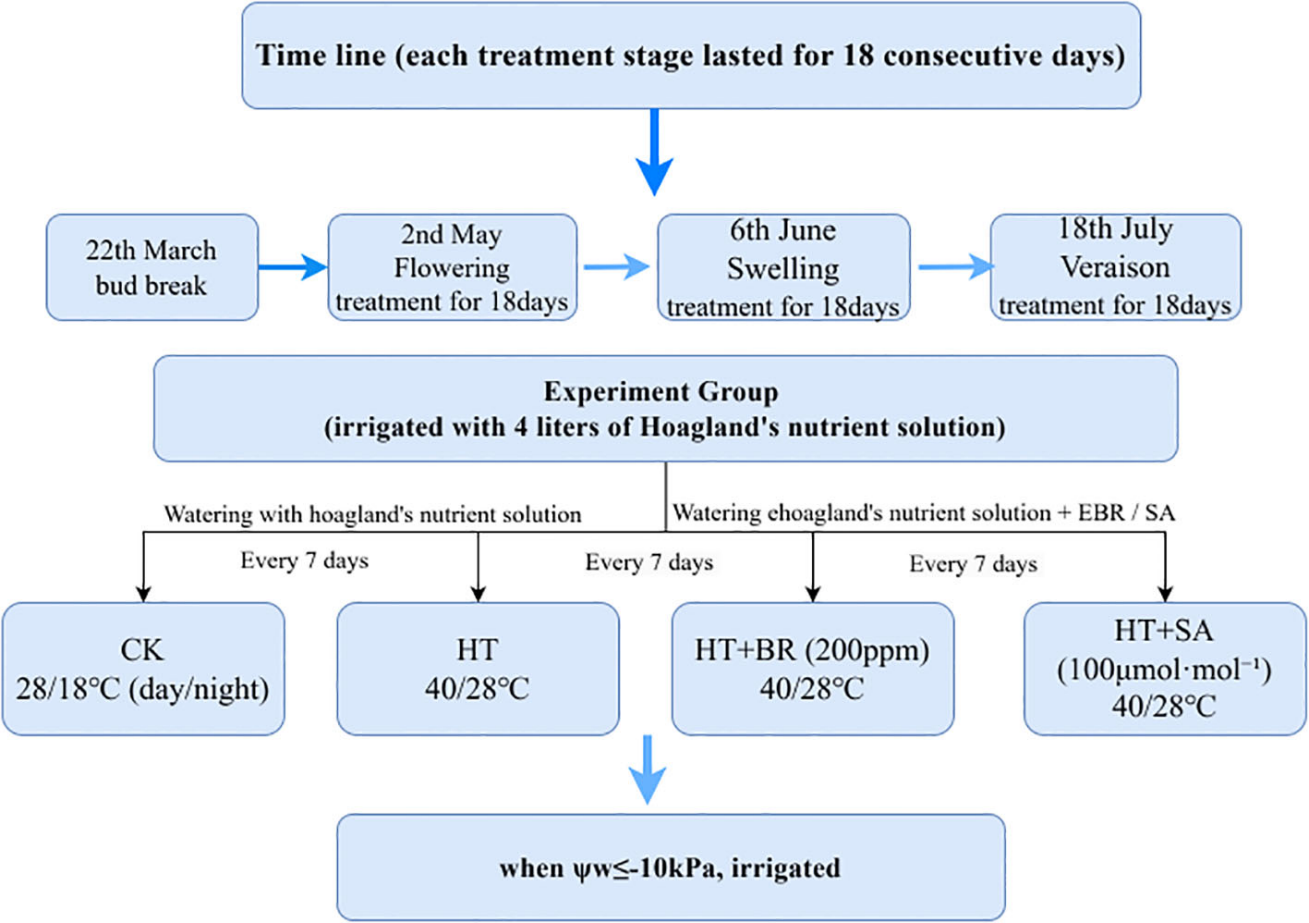
Experimental schematic diagram.

Three-year-old potted ‘Kyoho’ grapevines were utilized as test materials in this study. In January 2021, the grapevines were pruned and transplanted into 46.5-liter plastic pots containing a 1:1:1 (v/v/v) mixture of loam, organic fertilizer, and perlite. Each pot was equipped with two drip irrigation lines for nutrient solution delivery. The plants were maintained in a greenhouse at Shanghai Jiao Tong University, Shanghai, China (31°11′ N, 121°29′ E), arranged with 100 cm plant spacing and 200 cm row spacing. Budburst occurred on March 22, followed by flowering on May 2, with each vine producing four shoots and four flower clusters. Hoagland’s nutrient solution (120 ppm) was applied at a rate of 4 liters per pot when the soil water potential reached φw ≤ -10 kPa. Before flowering, 80 grapevines with uniform growth were selected and randomly assigned to three treatment stages: flowering, young berry swelling, and veraison. Each treatment stage lasted 18 consecutive days, commencing on May 2 (full bloom), June 6 (swelling stage), and July 18 (veraison), respectively. Prior to the heat treatments, the grapevines were acclimated for two days in a phytotron under controlled conditions of 65–75% relative humidity and a 25°C/18°Cday/night temperature cycle. Air temperature and humidity were monitored using HOBO U23-002 data loggers (Onset Computer Corporation, Cape Cod, USA). In April, a preliminary experiment was conducted on three-year-old ‘Kyoho’ grapevines to determine the optimal hormone concentrations (**Supplementary Table 1**). The results showed that the ‘Kyoho’ grape seedlings grew well in solutions of 100 μmol·L^-^¹ SA, and 200ppm 24-epibrassinolide (EBR). Based on preliminary tests assessing the effects of different concentrations of BR and SA on grape seedling growth, the final concentrations of 200 ppm EBR and 100 μmol·L^-^¹ SA were selected for the experiment. The study involved four groups of grapevines, with five replicates in each group. In the Heat Treatment (HT) group (Sreedharan, Philip et al.), the grapevines were exposed to high daytime temperatures exceeding 40°C (9:00–18:00) and cooler nighttime temperatures below 28°C (18:00–9:00). The Control (CK) group, by contrast, was kept under moderate conditions, with daytime temperatures kept below 30°C and nighttime temperatures above 20°C. The Heat Treatment + BR (HT+BR) group faced the same heat stress conditions as the HT group but received Hoagland’s nutrient solution supplemented with 200 ppm of 24-epibrassinolide (EBR). Similarly, the Heat Treatment + SA (HT+SA) group also experienced heat stress but was treated with Hoagland’s nutrient solution containing 100 μmol·L^-^¹ of salicylic acid (SA). All grapevines were initially irrigated with 4 liters of Hoagland’s nutrient solution (120 ppm nitrogen). Subsequent watering occurred every 7 days, with the BR and SA groups receiving their respective solutions. Additional irrigation was provided as needed when the soil water potential dropped to -10 kPa (**Figure 1**). During the flowering, young berry swelling, and veraison stages, gas exchange parameters, chlorophyll fluorescence parameters, and chlorophyll content were measured on the 6th to 7th leaves from the base of the new shoots. Leaf samples were collected from equivalent positions on new shoots to maintain consistency and were frozen for subsequent analysis. Three replicates were taken for each treatment.

### Gas-exchange measurements

Photosynthetic parameters were assessed on fully expanded leaves located in the mid-shoot region (node positions 4–5) using a CIRAS-3 Portable Photosynthesis System (PP Systems, Amesbury, USA) equipped with a PLC3 Universal Leaf Cuvette (6 cm² area). The measurements were conducted under controlled conditions with an ambient CO_2_ concentration of 400 μmol·mol^-^¹ and an airflow rate of 420 mmol·m^-^²·s^-^¹. Parameters recorded included net CO_2_ assimilation rate (An), stomatal conductance (gs), transpiration rate (E), and the leaf-to-air vapor pressure deficit (VPD). To evaluate the light response of photosynthesis, measurements were taken from 8:00 a.m. to 11:00 a.m. on the 10th day of treatment. Leaves were exposed to a series of light intensities (0, 50, 100, 200, 500, 800, 1000, 1200, 1500, 1800, and 2000 μmol photons·m^-^²·s^-^¹), with readings collected after 10 seconds of stabilization at each intensity. These data were used to generate light-response curves following the method described by Ye et al. (2013). Key photosynthetic parameters were derived, including the apparent quantum yield of photosynthesis (AQY), maximum net photosynthetic rate (PNmax), dark respiration rate (Rd), photoinhibition coefficient (βp), and a light saturation coefficient (γp). These calculations were performed using an online model developed by Ye (2010) (http://photosynthetic.sinaapp.com/). On the 13th day of treatment, CO_2_ response curves were generated for the same leaves during the same morning timeframe (8:00 a.m. to 11:00 a.m.). Measurements were taken across a range of CO_2_ concentrations (0 to 1500 μmol·mol^-^¹), allowing the net photosynthetic rate (PN) to stabilize at each level before recording. The CO_2_ response data were analyzed using the method outlined by Ye (2010) to estimate carboxylation efficiency (CE), maximum photosynthetic rate under CO_2_ saturation (Amax), and the photorespiration rate (Rp). These calculations were also performed using the same online photosynthetic modeling platform.

### Chlorophyll fluorescence parameter measurement

Chlorophyll fluorescence parameters were measured at room temperature using a Plant Efficiency Analyzer (PEA) (Hansatech, England). Leaves were placed into a black acetal adapter to ensure complete dark adaptation for 30 minutes prior to measuring the initial fluorescence (F_o_). An excitation light of 650 nm with an intensity of 3000 μmol·m^-^²·s^-^¹ was used to generate maximal fluorescence (F_m_). The maximum quantum yield of PSII (Fv/Fm) was calculated using the formula:


$$\frac{Fv}{Fm}=\frac{Fm-Fo}{Fm}$$

### Method for fruit set rate calculation

Prior to flowering, fruit clusters were bagged to protect them from environmental interference. After the 18-day high-temperature stress treatment during the flowering stage, the bags were removed. The number of fallen flowers was manually counted and compared with the number of young fruits on the clusters to calculate the fruit set rate.

### Determination of physiological parameters

The malondialdehyde (MDA) content was determined using the trichloroacetic acid (TCA) method (Hodges et al., 1999), while soluble protein content was quantified using Coomassie Brilliant Blue G-250. Both parameters were measured with a portable spectrophotometer (Linshang, CHN). Relative electrolyte leakage (REL) was measured based on the protocol described by Liu et al. (2023), with slight modifications. For REL measurement, leaf samples were collected and transported to the laboratory in darkness. Leaves were rinsed with clean water, washed three times with ultrapure water, and dried using filter paper. They were then cut into small pieces and placed in tubes containing 25 mL of deionized water. After incubating the samples in darkness for 12 hours, the initial conductivity (E₁) was measured using a conductivity meter (Leizi, Shanghai, China). The tubes were then heated to 100°C for 25 minutes, and the final conductivity (E_2_) was recorded after cooling. REL was calculated using the formula:


$$\text{REL}\%=\frac{\text{E}1}{\text{E}2}\times 100$$

Soluble protein (SP) content was determined by measuring absorbance at 595 nm using Bradford (1976), with bovine serum albumin as the standard. Results were expressed as mg·g^-^¹ fresh weight (FW).

### Determination of quality-related parameters

At each sampling stage, twelve berries were randomly selected per grapevine for each biological replicate. Total soluble solids (TSS) and titratable acidity (TA) were measured at 7-day intervals between 77 and 106 days after anthesis (DAA) using a saccharimeter (OWELL, Hangzhou, CHN) and a potential titration meter (HAINENG, Shenzhen, CHN), respectively. Berry dimensions, including longitudinal and equatorial diameters, were measured using a vernier caliper (Mitutoyo, Tokyo, Japan), while berry weight was recorded using an analytical balance (Sartorius, Germany). Total anthocyanin content was determined using a portable spectrophotometer (Linshang, CHN), following the method of Li D et al. (2021). Glucose and fructose levels were analyzed using high-performance liquid chromatography (HPLC) based on the protocol by Li et al. (2021). To prepare samples, 1 mL of grape juice was diluted with 9 mL of deionized water, filtered through a 0.22 μm membrane, and analyzed using an HPLC system (LC3000, CXTH, Beijing, CHN) equipped with a ZORBAX NH_2_ chromatographic column (250 × 4.6 mm, 5 μm, Agilent). The mobile phase consisted of an 80% acetonitrile aqueous solution, with a flow rate of 0.75 mL·min^-^¹, a column temperature of 25°C, and an injection volume of 20 μL. Glucose and fructose concentrations were calculated based on standard curves generated from the peak areas of the chromatographic profiles. Each analysis was conducted in triplicate for biological replicates.

### Statistical analysis

Data analysis was conducted using Excel 2019, and all experiments were independently repeated three times. The results are presented as the mean values with standard errors. Two-way ANOVA assessed treatment effects and time responses, significant at p< 0.05. Duncan’s test (p< 0.05) compared treatment means. Student’s t-tests compared photosynthesis and gas exchange parameters, with Bonferroni correction for multiple comparisons. Origin 9.0 created all figures, aiding data visualization and treatment effect comparison.

## Results

### Temperature monitoring during high-temperature treatment

**Figure 2:**
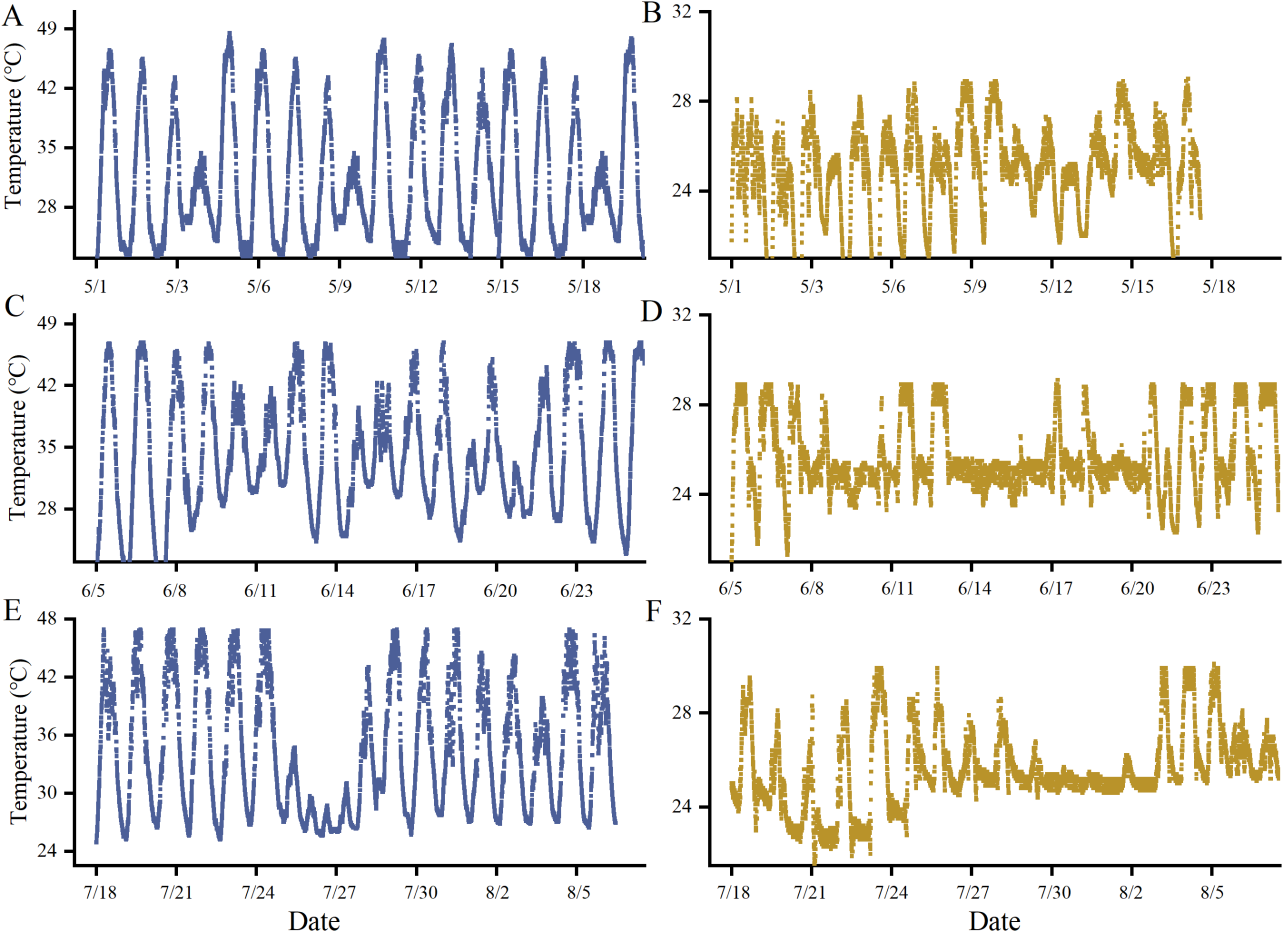
Temperature monitoring at three developmental stages of grapevines during heat stress. [**(A–C)** show the temperature during flowering, young berry swelling, and veraison in the heat stress phytotron, respectively; **(D–F)** show the temperature during flowering, young berry swelling, and veraison in the control phytotron, respectively].

During the three stages of high-temperature treatment, temperatures in the indoor phytotrons were carefully monitored to ensure consistency. In the high-temperature treatment chamber, maximum temperatures ranged from 40°C to 45°C, with an average daytime temperature of 40°C and an average nighttime temperature of 28°C. In contrast, the control chamber maintained maximum temperatures between 28°C and 30°C, with an average daytime temperature of 28°C and an average nighttime temperature of 18°C. The temperature conditions in the high-temperature treatment chamber closely resembled those typically observed in glass greenhouses during periods of high-temperature weather (**Figure 2**).

### Effects of exogenous compounds on photosynthesis, and Fv/Fm of ‘Kyoho’ leaves under heat stress

**Figure 3:**
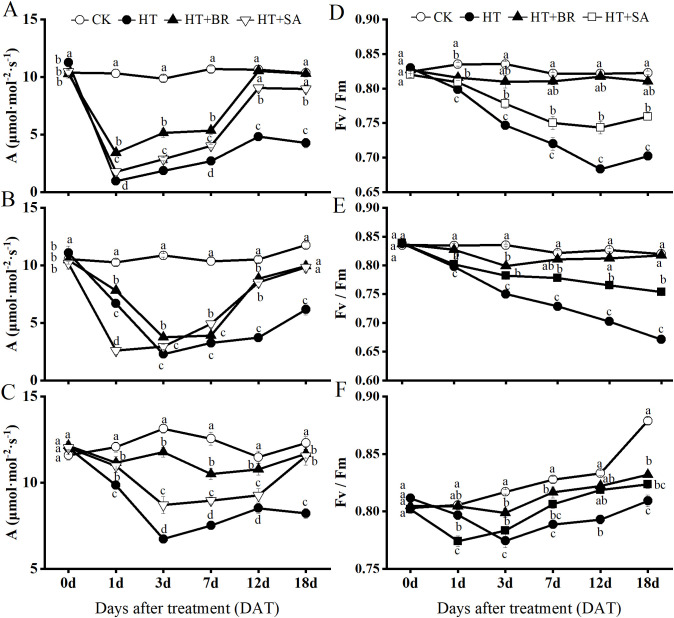
Effects of heat stress and exogenous substances on the photosynthetic efficiency of ‘Kyoho’ grape leaves. (**(A–C)** represent the maximum photosynthetic rate during flowering, young berry swelling, and veraison, respectively; **(D–F)** represent the maximal PS II efficiency (Fv/Fm), respectively) Vertical bars represent the standard deviation (n = 6). Values with different lowercase letters with the same sampling date are significantly different according to Duncan’s multiple range test (DMRT) at the 5% level.

Heat stress significantly impacted the maximum photosynthetic rate (Amax) of ‘Kyoho’ grape leaves across the flowering, young berry swelling, and veraison stages (**Figure 3**). During the flowering stage (**Figure 3A**), Amax in the high-temperature (HT) group dropped dramatically to 9.21% of the control (CK) after just one day of heat stress. However, the application of BR and SA mitigated the damage caused by heat stress to varying degrees, with BR showing a more pronounced effect. By the 12th day, the Amax in the BR+HT group approached levels similar to the control. On the 18th day, the Amax values for the BR and SA groups were 96.14% and 86.64% of the control, respectively.

In the young berry swelling and veraison stages, the photosynthetic rate in the HT group remained significantly lower than that of the CK group. However, Amax values during these stages were notably higher than those recorded during the flowering stage, suggesting an increase in heat tolerance as the plants developed (**Figures 3B, C**). Root application of BR and SA continued to alleviate the effects of heat stress during these stages, with both treatments showing significant protective effects. By the 18th day of treatment, Amax values in the BR and SA groups were approximately 95% and 93.77% of the control, respectively. These findings indicate that BR and SA significantly enhance photosynthetic rates under heat stress, with their effects becoming more pronounced as the grapevines matured.

The Fv/Fm parameter, which reflects the photochemical efficiency of photosystem II (PSII) and the overall physiological status of the plant, also demonstrated significant trends during heat stress. During the flowering and young berry swelling stages, the CK group maintained stable photosynthetic efficiency, with minimal changes in Fv/Fm values (**Figures 3D, E**). In contrast, heat stress caused a significant reduction in Fv/Fm across all other treatment groups. Nevertheless, BR and SA treatments effectively mitigated these declines, helping to preserve PSII functionality. During the veraison stage, the Fv/Fm values in the CK group gradually increased, reflecting enhanced photosynthetic activity as the leaves worked to produce more organic compounds needed for anthocyanin accumulation (**Figure 3F**). The trend in Fv/Fm for the BR group closely paralleled that of the CK group, with only a slight deviation observed on day 3.

Overall, the exogenous application of BR and SA demonstrated significant restorative effects on the photosynthetic efficiency of ‘Kyoho’ grape leaves across all three phenological stages. These treatments mitigated the adverse effects of heat stress, promoted recovery of PSII activity, and improved overall photosynthetic performance, particularly during the later stages of grape development.

### Effects of exogenous compounds on the light response (A-PPFD) and CO_2_ curves (A -Ci)

**Figure 4:**
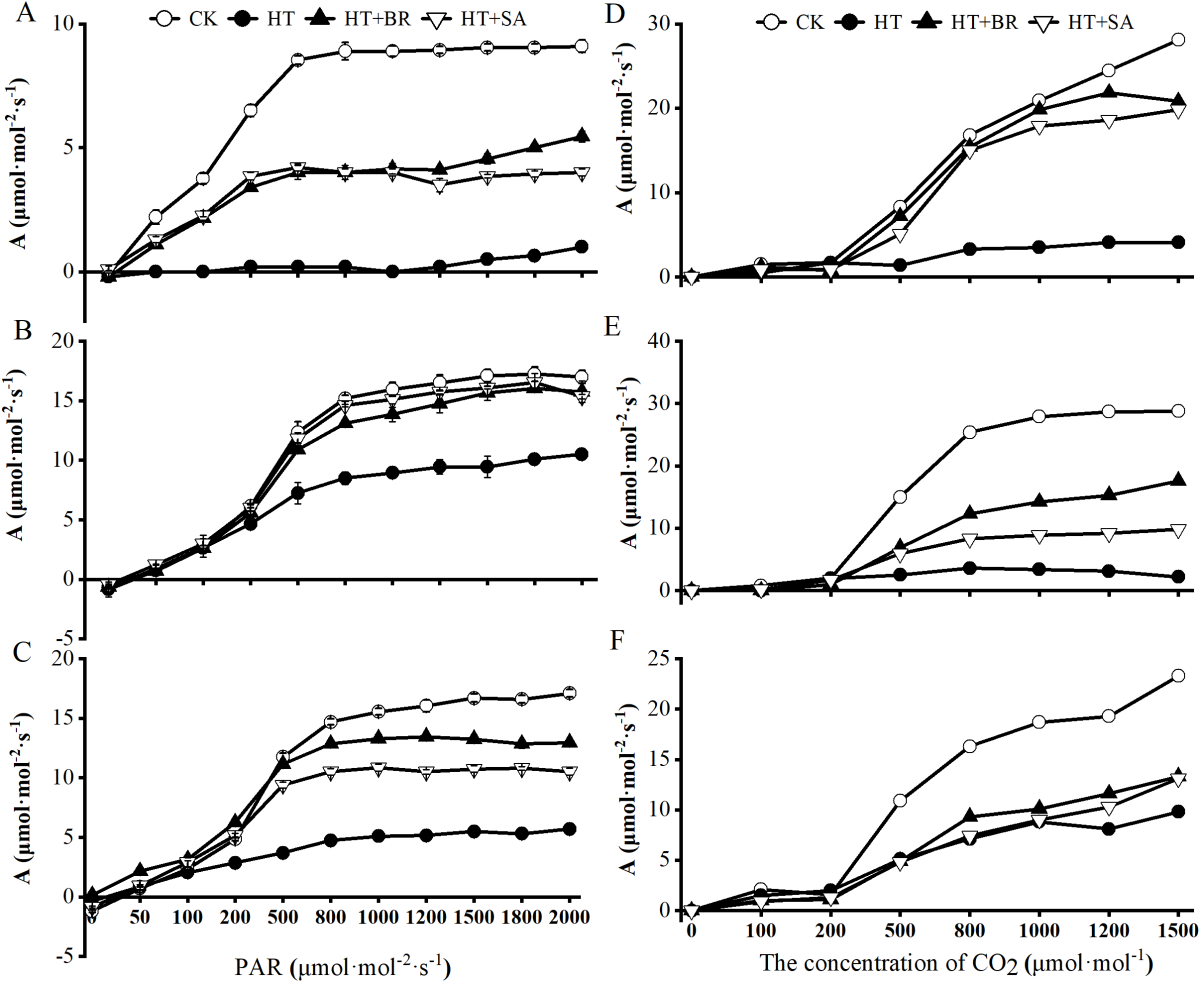
Effects of heat stress and exogenous substances on light- and CO_2_-response curves of photosynthesis in ‘Kyoho’ grape leaves at different developmental stages. (**(A–C)** represent the maximum photosynthetic rates during flowering, young berry swelling, and veraison for the light-response curve, respectively; **(D–F)** show the corresponding rates for the CO_2_-response curves, respectively).

To investigate how heat stress and exogenous compounds affect photosynthesis during flowering, young berry swelling, and veraison, we measured the light-response of photosynthesis in ‘Kyoho’ grape leaves. Net photosynthetic rate (Pn) showed a different trend in three periods with increasing photosynthetic photon flux density (PPFD). In three stages, the PPFD where the PN became closely saturated with incremental increase of light intensity was different under different conditions. The inflection points of the CK, HT+BR and HT+SA treatments at flowering stage occurred earlier than that of other stages. Moreover, the Pn of the HT group was almost zero when PPFD was below 1200 μmol·m^-^²·s^-^¹, and only increased once it reached 1200 μmol·m^-^²·s^-^¹. This indicates that the leaves of ‘Kyoho’ plants under heat stress during flowering are unable to perform effective photosynthesis at light intensities below 1200 μmol·m^-^²·s^-^¹ in flowering stage (**Figure 4A**). During the young berry swelling stage (**Figure 4B**), the differences in Pn among the treatments decreased. At saturation light intensity, the Pn ranking was as follows: CK > BR ≈ SA > HT. The HT treatment reached its light saturation point at 500 μmol·mol^-^²·s^-^¹, whereas the other treatments achieved their maximum light saturation points approximately at 1200 μmol·mol^-^²·s^-^¹. At this saturation light intensity, the Pn values for the BR and SA groups were 92.65% and 90.29% of the CK, respectively, while the HT group exhibited only 61.76% of the CK’s Pn. This suggests that the foliar application of BR and SA significantly improved leaf photosynthetic performance during the young berry swelling stage. During veraison (**Figure 4C**), the A-PPFD curve followed a pattern similar to that observed during the young berry swelling stage. At a PPFD of 2000 μmol·m^-^²·s^-^¹, the Pn ranking was as follows: CK > BR > SA > HT. Throughout the light response, the exogenous BR and SA treatments showed relatively stable recovery of photosynthetic activity under heat stress.

The Pn-Ci curve provides a direct and effective method for assessing Rubisco activity, RuBP carboxylation, triose phosphate utilization, and photorespiration. Under high-temperature stress, plants in the HT group exhibited greater susceptibility to CO_2_ saturation, whereas plants in the BR and SA treatment groups showed the opposite trend. This suggests that exogenous applications of BR and SA through root irrigation can significantly enhance CO_2_ fixation rates under heat stress. Furthermore, at all three developmental stages of grapes, when CO_2_ concentration reached 1500 μmol·mol^-^¹, the Pn in the HT group was only 7.6% to 14.6% of that in CK, whereas Pn in the BR and SA-treated leaves reached 35% to 74% of the CK values, significantly improving the CO_2_ fixation capacity of leaves under heat stress (**Figures 4D-F**).

Analysis of the calculated values from the Pn-PPFD curve revealed that BR and SA significantly influenced the photosynthetic process under heat stress conditions. Across the three critical developmental stages, heat stress (HT) notably reduced the maximum net photosynthetic rate (Pnmax) and increased the light inhibition coefficient (βp). During the flowering and young berry swelling stages, the light saturation coefficient (γp) in the HT group was -0.09 and -0.07, respectively, indicating a negative effect of high temperatures. However, during the veraison stage, γp shifted to positive values, which may be attributed to the combined effects of reduced leaf senescence and increased chlorophyll content. Remarkably, BR and SA treatments significantly improved Pnmax and γp under heat stress conditions, underscoring their ability to enhance leaf resistance to high temperatures. These results highlight the protective role of BR and SA in sustaining photosynthetic performance under environmental stress. Further analysis based on the Pn-Ci curve showed that HT significantly reduced carboxylation efficiency (CE), maximum photosynthetic rate (Amax), and photorespiration rate (Rp). Conversely, BR and SA treatments markedly increased Amax and Rp under HT conditions, demonstrating their effectiveness in mitigating the adverse impacts of heat stress on photosynthesis. These findings further reinforce the potential of BR and SA to improve the photosynthetic capacity of ‘Kyoho’ grape leaves and alleviate the detrimental effects of heat stress (**Table 1**).

**Table 1:** Fitted photosynthetic parameters via light- or CO_2_-response curves under different light and temperature conditions.

Fitted parameter		Flowering stage				Young berry swelling				veraison			
		CK	HT	HT+BR	HT+SA	CK	HT	HT+BR	HT+SA	CK	HT	HT+BR	HT+SA
Light-response	AQY	0.031	0	0.021	0.192	0.034	0.015	0.033	0.035	0.036	0.024	0.030	0.038
	Pnmax (μmolm^-2^s^-1^)	9.07	1.00	5.85	4.09	17.22	4.03	15.92	16.20	16.93	5.70	13.58	10.96
	Rd (μmol m^-2^ s^-1^)	0.261	0.200	0.292	0.320	1.090	-0.200	0.992	0.664	1.418	0.388	0.382	1.060
	*β*_p*_10^-3^ (m^-2^ s^-1^)	0.119	0.285	0.167	0.148	0.147	0.419	0.086	0.173	0.151	-0.070	0.187	0.142
	*γ*_p *_ 10^-3^ (m^-2^ s^-1^)	5.41	-0.09	14.50	9.05	1.36	-0.07	1.67	1.23	1.18	6.26	1.67	2.66
	R^2^	0.995	0.991	0.996	0.980	0.999	0.989	0.999	0.999	0.999	0.995	0.999	0.997
CO_2_-response	CE	0.023	0.004	0.027	0.022	0.044	0.007	0.019	0.015	0.025	0.011	0.013	0.010
	Amax (μmolm^-2^s^-1^)	28.10	4.46	23.58	23.50	29.54	3.49	19.66	6.70	24.44	9.61	16.15	14.15
	Rp (μmolm^-2^s^-1^)	1.25	-0.41	2.25	1.78	3.09	0.12	1.41	0.65	0.90	-0.94	0.54	0.16
	R^2^	0.994	0.989	0.991	0.992	0.994	0.993	0.995	0.991	0.998	0.990	0.990	0.997

AQY, apparent quantum yield of photosynthesis; Pnmax, maximum net photosynthetic rate; Rd, dark respiration rate; βp, light inhibition coefficient; γp, light saturation coefficient; CE, carboxylation efficiency; Amax, maximum photosynthetic rate at CO_2_ saturation; Rp, Photorespiration rate. R^2^, Determination coefficient.

### Impacts of exogenous compounds on the antioxidant activity of leaves under heat stress

**Figure 5:**
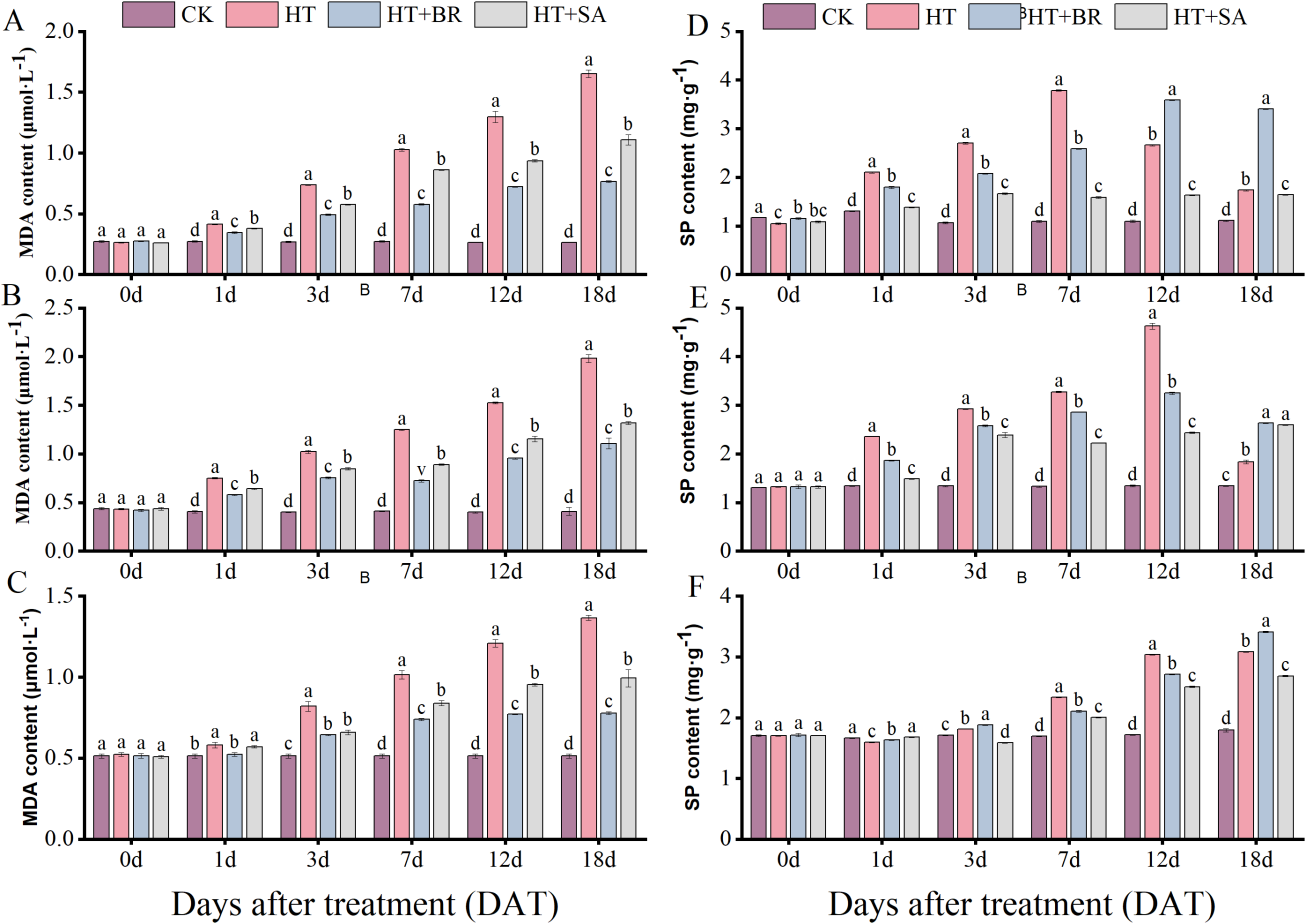
Effects of heat stress and exogenous substances on MDA and soluble protein contents in ‘Kyoho’ leaves. (**(A–C)** represent changes in malondialdehyde content; **(D–F)** represent changes in soluble protein content in leaves at flowering, young berry swelling, and veraison, respectively). Vertical bars represent standard deviations (n = 3). Values with different lowercase letters with the same sampling date are significantly different according to Duncan’s multiple range test (DMRT) at the 5% level.

Malondialdehyde (MDA) and soluble protein (SP) content are widely used as indicators for assessing plant stress resistance, providing insights into the extent and severity of stress damage. During the three developmental stages, MDA levels in the CK group remained relatively stable, reflecting the integrity and stability of the cell membrane under normal conditions (**Figures 5A–C**). In contrast, MDA content in the HT group progressively increased throughout the stages, reaching 6.23, 2.88, and 4.17 times the levels observed in the CK group by the end of the flowering, young berry swelling, and veraison stages, respectively.

The application of BR and SA through root irrigation significantly alleviated the effects of heat stress, with BR demonstrating the most pronounced impact, followed by SA. By the end of the three treatment periods, MDA content in the BR-treated group was reduced to approximately 46%, 56%, and 57% of the levels recorded in the HT group during the flowering, young berry swelling, and veraison stages, respectively. These findings highlight the effectiveness of BR and SA in mitigating membrane damage caused by heat stress.

Soluble protein (SP) content in the leaves increased significantly under high-temperature stress across all three developmental stages. Both BR and SA treatments partially mitigated this increase, but their effects were primarily evident during the initial 7 days of flowering and the first 12 days of the fruit expansion and veraison stages. Beyond these time points, the prolonged effects of high-temperature stress proved difficult to counteract through these treatments (**Figures 5D–F**). These results suggest that while BR and SA can provide initial protection and reduce heat stress damage, prolonged exposure to high temperatures can surpass the reparative capacity of these treatments.

**Figure 6:**
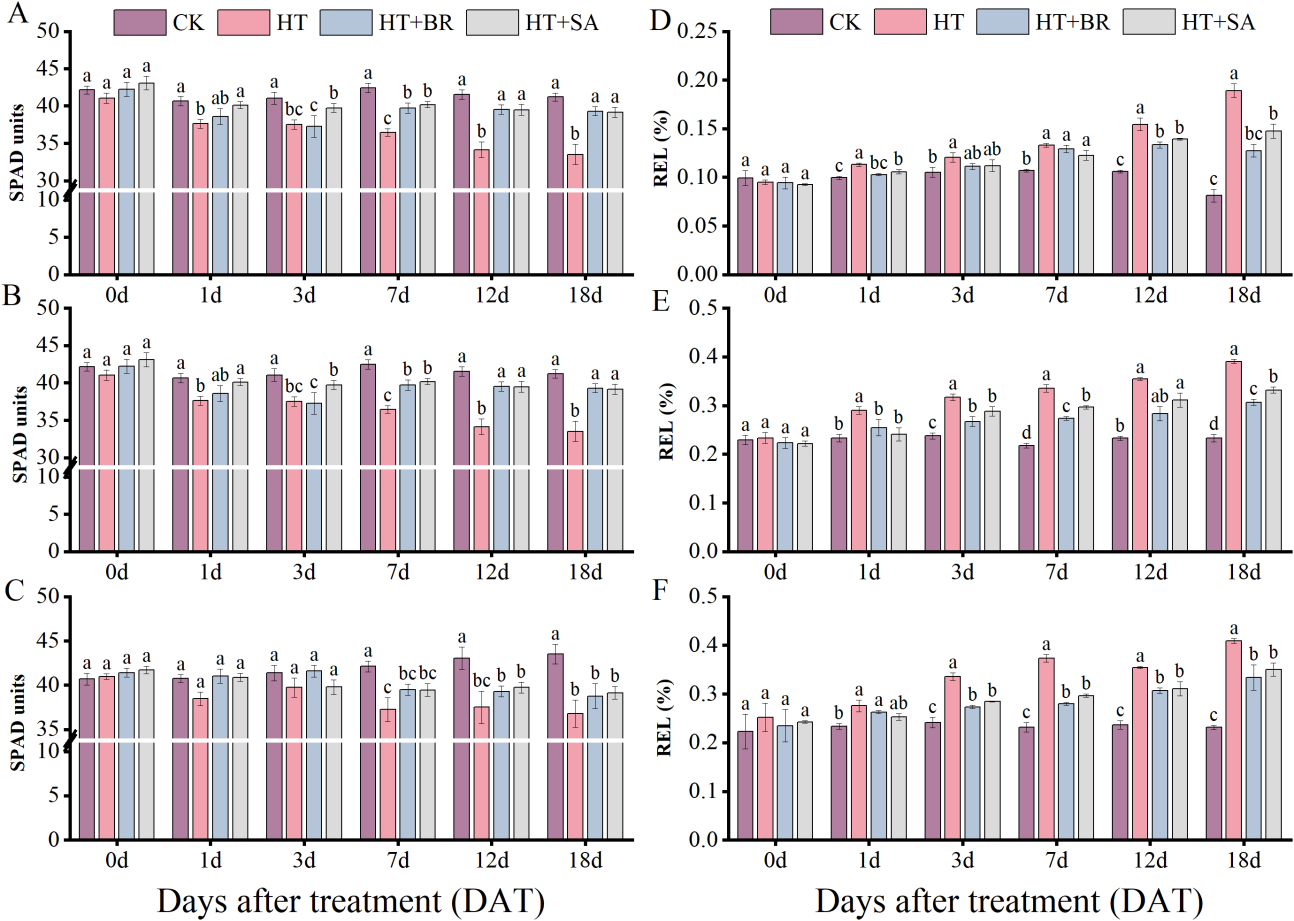
Effects of heat stress and exogenous substances on chlorophyll relative content and electrical conductivity in ‘Kyoho’ grape leaves. (**(A–C)** represent changes in chlorophyll relative content; **(D–F)** represent changes in leaf electrical conductivity at flowering, young berry swelling, and veraison, respectively.) Vertical bars represent standard deviations (n = 9). Values with different lowercase letters with the same sampling date are significantly different according to Duncan’s multiple range test (DMRT) at the 5% level.

To investigate the effects of exogenous substances on grape leaves under heat stress, we measured leaf relative chlorophyll content (SPAD) and relative electrical conductivity (REL) (**Figure 6**). Heat stress caused a reduction in SPAD values across all three developmental stages (**Figures 6A–C**). However, short-term heat exposure did not significantly affect SPAD values. During the flowering and young berry swelling stages, exogenous applications of BR and SA significantly alleviated chlorophyll degradation caused by heat stress, with notable improvements observed from 3 days after treatment (3 DAT). At the veraison stage, no significant differences in chlorophyll content were observed among the treatments after heat stress, which may be attributed to leaf aging. Older leaves tend to exhibit enhanced stress resistance compared to younger leaves. Despite this, the chlorophyll content in the HT group declined significantly and showed no signs of recovery. By 18 DAT, the chlorophyll content in the HT group was only 86.3% of the CK value, a statistically significant reduction when compared to the BR and SA treatment groups (P<0.05). The relative electrical conductivity (REL) of plant leaves serves as an indicator of cell membrane permeability, reflecting the degree of damage caused by heat stress. Under heat stress, REL values increased progressively with the duration of stress (**Figures 6D–F**). During the flowering stage, BR and SA treatments had a relatively limited effect in alleviating the impacts of short-term heat stress, with no significant differences between the exogenous treatment groups and the CK at 18 DAT. However, during the young berry swelling and veraison stages, BR and SA treatments demonstrated more pronounced improvements in REL under high-temperature conditions. BR showed a stronger effect on maintaining membrane stability and reducing permeability compared to SA.

### Impacts of exogenous compounds on fruit appearance index under heat stress

**Figure 7:**
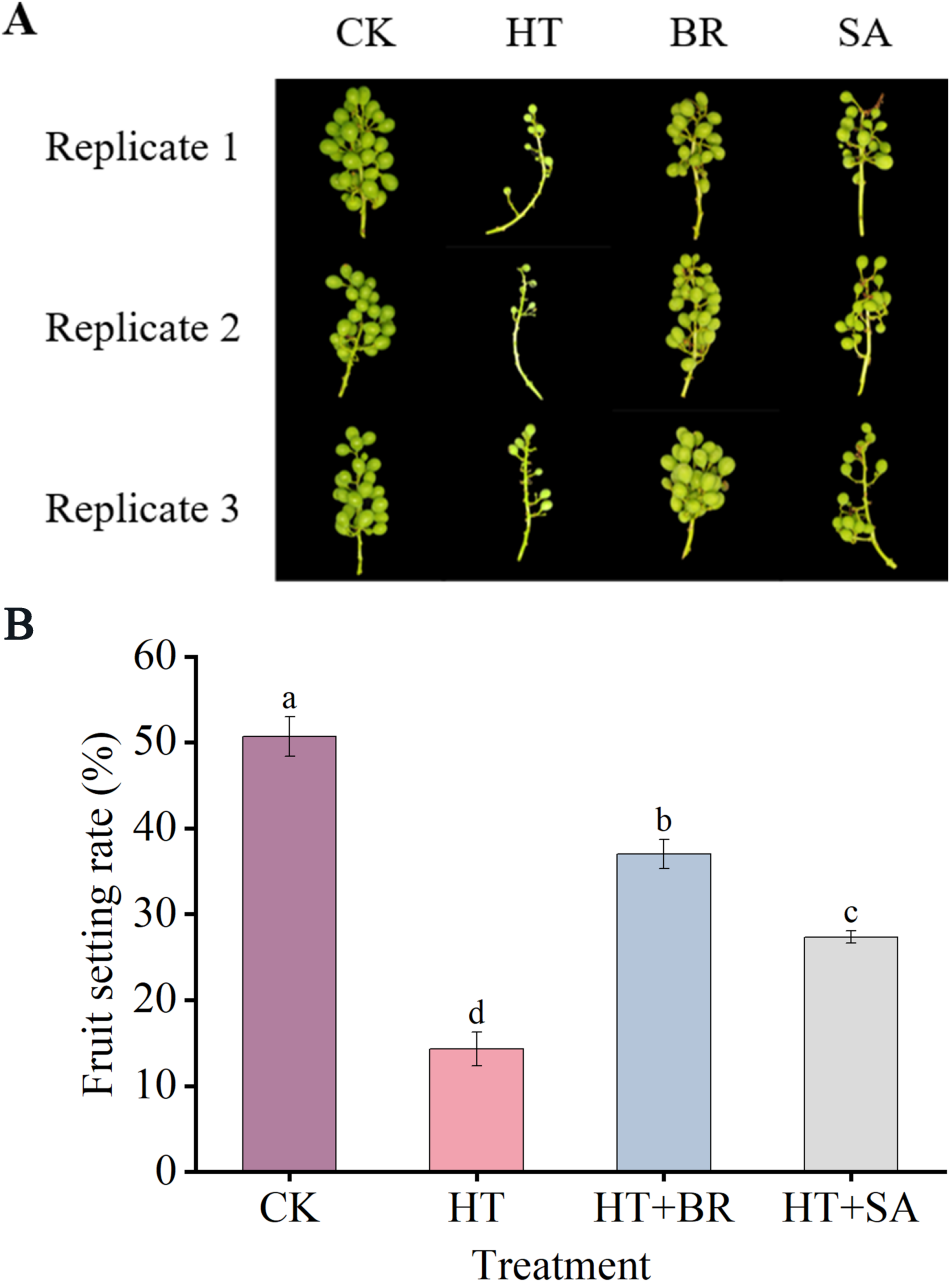
Effects of heat stress and exogenous substances on the berry setting rate of ‘Kyoho’ grapes. **(A)** shows the phenotype diagram for each treatment at the end of flowering, and **(B)** shows the comparison of berry setting rates for each treatment at the end of flowering. Vertical bars represent standard deviations (n = 3). Values with different lowercase letters with the same sampling date are significantly different according to Duncan’s multiple range test (DMRT) at the 5% level.

To further examine the effects of exogenous compounds on berry development and quality under heat stress, the fruit set rate of each treatment group during the flowering period was recorded (**Figure 7**). Heat stress significantly impaired the fruit-setting ability of ‘Kyoho’ grapes, reducing the fruit set rate from 50.1% in the CK group to 14.3% in the HT group. The application of BR substantially improved the fruit set rate, increasing it to 37%. While SA treatment also enhanced the fruit set rate under heat stress, its effect was less pronounced compared to BR.

**Figure 8:**
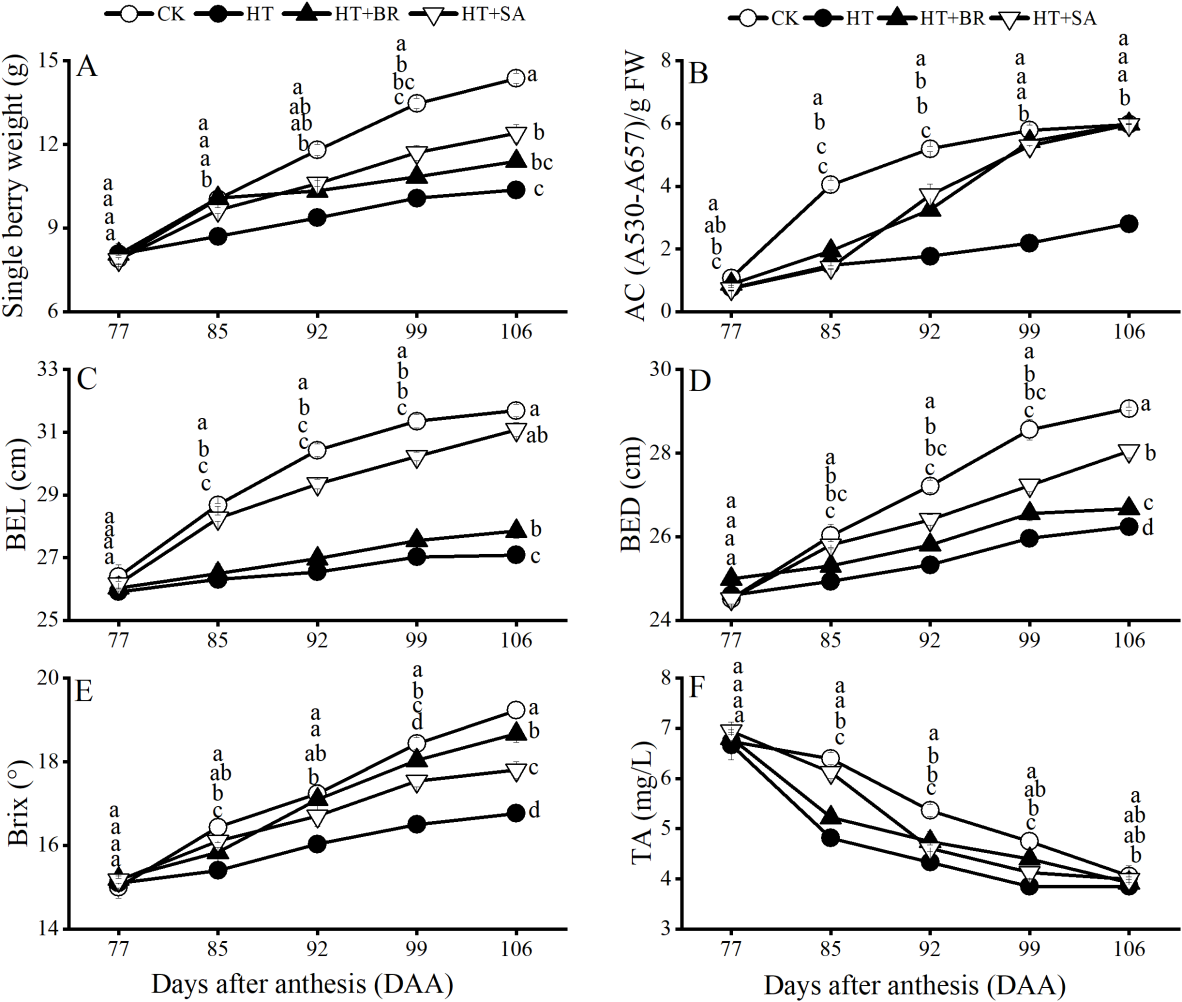
Effects of heat stress and exogenous substances on the developmental trends of berry quality in ‘Kyoho’ grapes from veraison to ripening. **(A–F)** represent the single berry weight, anthocyanin content, berry equatorial diameter, berry vertical diameter, brix, and titratable acid content in the pericarp of each treatment from veraison to ripening, respectively. Vertical bars represent standard deviations (n = 3). Values with different lowercase letters on the same sampling date are significantly different according to Duncan's multiple range test (DMRT) at the 5% level.

The single berry weight, berry longitudinal diameter (BLD), berry equatorial diameter (BED), total soluble solids (TSS), titratable acidity (TA), and anthocyanin content (AC) of the fruit were measured (**Figure 8**). Heat stress significantly reduced single berry weight, BLD, and BED in the HT group compared to the CK group. Exogenous treatments with SA and BR effectively alleviated these adverse effects to varying degrees, with SA demonstrating a particularly notable impact (**Figures 8A–C**). In terms of fruit quality, heat stress severely impaired the accumulation of TSS and anthocyanins, with the AC at maturity reaching only 46.8% of that in the CK group. However, both BR and SA treatments promoted anthocyanin accumulation under heat stress to varying extents (**Figure 8F**). Additionally, BR significantly enhanced TSS accumulation compared to the HT group (**Figure 8E**). Although high temperatures accelerated the decline in fruit TA, neither BR nor SA treatments had a significant influence on TA metabolism.

### Effects of exogenous compounds on monosaccharide components

**Figure 9:**
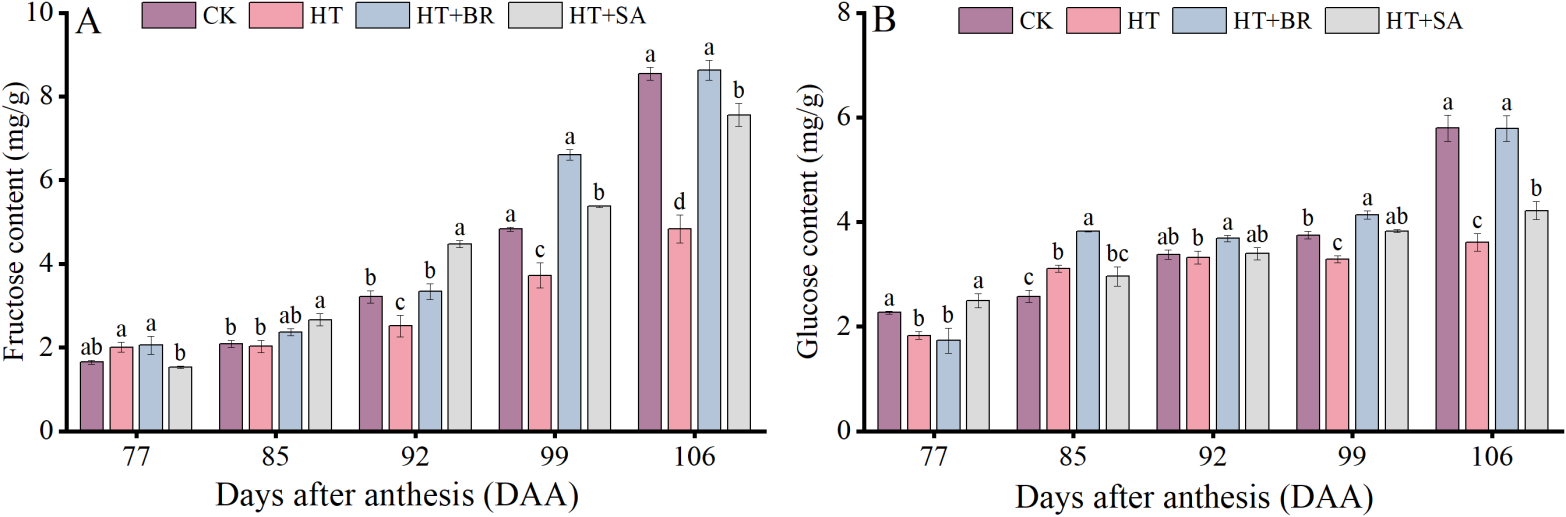
Effects of exogenous substances on the monosaccharide components [**(A, B)** represent the fructose and glucose contents, respectively]. Vertical bars represent standard deviations (n = 3). Values with different lowercase letters with the same sampling date are significantly different according to Duncan’s multiple range test (DMRT) at the 5% level.

To further examine the effects of exogenous compounds on fruit quality under heat stress, we measured changes in monosaccharide composition during berry development (**Figure 9**). Heat stress significantly inhibited the accumulation of fructose and glucose in the berries, with their contents reaching only 56.6% and 62.4% of those in the CK group at 106 days after anthesis (DAA), respectively. However, treatments with BR and SA improved the accumulation of fructose and glucose under heat stress to varying degrees. Among these, BR demonstrated the most pronounced effect, while the impact of SA was comparatively weaker.

## Discussion

Increasing global temperatures due to climate change significantly threaten agricultural productivity. Even with ventilation and cooling systems, excessive heat during summer continues to impair fruit coloration and quality. To understand the impact of heat stress on ‘Kyoho’ grapes, we simulated an 18-day heat stress period (Tmax: 40°C, Tmin: 28°C; control: 28°C/18°C) across three developmental stages. Furthermore, we investigated the roles of BR and SA treatments in promoting photosynthetic growth and mitigating heat-induced damage. The findings revealed that BR and SA alleviate heat stress through distinct, stage-dependent mechanisms.

### Hormone treatment alleviates the inhibition of photosynthesis in leaves caused by heat stress

Photosystem II (PSII) is widely regarded as the most heat-sensitive component of photochemistry. Chlorophyll fluorescence has emerged as a reliable tool for detecting and quantifying heat stress-induced changes in PSII function (Lu and Zhang, 2000; Baker and Rosenqvist, 2004; Baker, 2008; Rizza et al., 2011). In this study, we utilized the Fv/Fm ratio to evaluate hormone-induced protection or enhancement of PSII under heat stress. In healthy plants, Fv/Fm values typically range from 0.75 to 0.85. However, under stress conditions, this ratio declines sharply, reflecting impaired photosynthetic performance (Kitajima and Butler, 1975; Baker, 2008).

Our results showed that the Fv/Fm values in the control group (CK) remained stable at approximately 0.82 across all developmental stages. In contrast, ‘Kyoho’ grapevines exposed to heat stress exhibited an immediate decrease in Fv/Fm values, indicating a decline in photosynthetic efficiency and highlighting the detrimental effects of high temperatures on grapevine growth (**Figures 3D, E**). During the flowering and young berry swelling stages, the Fv/Fm values in the CK group remained relatively stable, showing minimal variation. However, the Fv/Fm values in plants treated with exogenous hormones (BR and SA) under heat stress decreased significantly but were consistently higher than those in untreated heat-stressed plants. This suggests that BR and SA effectively mitigated PSII photoinhibition, enhancing its efficiency in CO_2_ assimilation and overall photosynthetic capacity, thereby improving the photosynthetic performance of ‘Kyoho’ grape leaves under high-temperature conditions.

Plant growth is closely tied to photosynthesis and respiration. In this study, heat stress significantly advanced the light saturation point of ‘Kyoho’ grape leaves. In the heat stress (HT) group, the net photosynthetic rate (Pn) remained near 0 μmol·m^-^²·s^-^¹ under increasing photosynthetic photon flux density (PPFD), with only a slight rise observed when PPFD exceeded 1200 μmol·m^-^²·s^-^¹. This indicates that grapevines exposed to heat stress during flowering were unable to conduct effective photosynthesis at light intensities below 1200 μmol·m^-^²·s^-^¹, thereby failing to accumulate sufficient carbohydrates to support the plant’s nutritional and growth demands. Photoinhibition, as indicated by the value of βp (Ye et al., 2013), was alleviated in the BR and SA treatment groups compared to the untreated control under heat stress (**Table 1**). These results demonstrate that BR and SA treatments mitigate photoinhibition under high-temperature conditions.

Heat stress caused a reduction in the light saturation point, while BR and SA treatments improved photosynthetic efficiency by enhancing Rubisco activity, which increased the light saturation point. Notably, when PPFD exceeded 200 μmol·m^-^²·s^-^¹, the growth rate of Pn in all treatment groups slowed (**Figures 4A-C**). At this stage, although both stomatal conductance and Pn increased, their rates of increase were not synchronized. High temperatures disrupted the coordination between CO_2_ assimilation and light absorption, impeded the transfer of photosynthetic electrons, and reduced Rubisco carboxylation activity, leading to a slower increase in Pn. Consequently, the HT group exhibited a significantly earlier saturation point and the lowest Pn values. Exogenous hormone treatments, especially BR and SA, alleviated the inhibition of photosynthesis caused by heat stress, improving overall photosynthetic performance under high-temperature conditions.

The CO_2_ response curve reflects the carboxylation efficiency of Rubisco, an essential enzyme in photosynthesis. Heat stress significantly reduced Rubisco’s carboxylation activity, as previously noted (Wang et al., 2010). The interplay between CO_2_ concentrations and photosynthetic and respiratory rates directly affects plant growth and yield, as greater CO_2_ assimilation enhances the accumulation of energy reserves, ultimately boosting growth and productivity (Bhargava and Mitra, 2021). In this study, the net photosynthetic rate (Pn) of all treatments increased with rising CO_2_ concentrations. However, under high-temperature (HT) stress, the CO_2_ saturation point was notably reduced. At a CO_2_ concentration of 1500 μmol·mol^-^¹, the Pn of the HT group was only one-seventh that of the control (CK) group. Maintaining a stable net CO_2_ assimilation rate is thus a reliable indicator of improved heat tolerance in plants. Temperatures exceeding optimal levels disrupt stomatal conductance, intracellular CO_2_ concentration, and leaf water status, leading to impaired photosynthesis (Carmo-Silva et al., 2012; Greer and Weedon, 2012). Stomatal closure under heat stress alters intracellular CO_2_ levels, further inhibiting net photosynthesis (Morales et al., 2003; Wahid et al., 2007). As a result, the HT group exhibited the lowest Pn values. Photosynthetic performance under high-temperature conditions is strongly influenced by the activation state of Rubisco. In this study, significant reductions in carboxylation efficiency (CE) were observed exclusively in the HT group across all three developmental stages (**Table 1**). This finding suggests that BR and SA treatments enhance plant resilience to heat stress by improving carboxylation efficiency and the regeneration of ribulose-1,5-bisphosphate (RuBP) (Yamori et al., 2006; Ogweno et al., 2008). The increase in βp due to heat stress suggests possible impairment to the photosynthetic apparatus. The reduction of βp values following BR and SA treatments implies that these hormones may offer protective effects against heat-induced damage to the photosystem (Kothari and Lachowiec, 2021). Fluctuations in the maximum photosynthetic rate (Amax) and the photorespiration rate (Rp) are indicative of a plant’s photosynthetic potential across varying CO_2_ levels. The enhancement of these parameters with BR and SA treatments suggests their role in bolstering the photosynthetic performance of plants, particularly under conditions of elevated CO_2_ concentrations (Zhang et al., 2021). Furthermore, the respiratory demand (RD), which reflects the consumption of photosynthetic products, was significantly higher in the BR and SA treatment groups, particularly during the fruit expansion and veraison stages. This indicates that these treatments enhanced the utilization of photosynthetic products in leaves under heat stress, promoting fruit growth and development (Hikosaka et al., 2006).

Plants have evolved complex mechanisms to mitigate the adverse effects of heat stress. Malondialdehyde (MDA), a terminal product of membrane lipid peroxidation, serves as a biomarker for oxidative damage severity under stress, with elevated levels directly signifying membrane integrity loss. Soluble proteins function as dual-purpose mediators, maintaining osmotic equilibrium through colloid protection while stabilizing enzymatic activity under thermal denaturation pressures. Heat stress disrupts PSII function, reduces photochemical efficiency, and generates reactive oxygen species (ROS), which compromise membrane permeability and cause lipid peroxidation, ultimately leading to cellular damage (Foyer and Shigeoka, 2011). In this study, MDA content in the HT group progressively increased throughout the treatment period. Soluble proteins, which play vital roles in maintaining cell osmotic potential and preventing dehydration, initially increased but later declined during flowering and berry swelling in response to heat stress (Zhao and Huang, 2021; Hong et al., 2023). This biphasic response may be attributed to the higher temperature (45°C) used in our study compared to other heat stress experiments (35–40°C). The initial increase in SP likely reflects osmotic regulation under acute stress, whereas the subsequent decline suggests protein degradation due to prolonged heat exposure. Interestingly, SP content continued to increase during the veraison stage, likely due to the plants’ adaptation to sustained high temperatures by this stage.

Exogenous treatments with BR and SA mitigated oxidative damage by reducing MDA levels and maintaining higher SP content. This protective effect improved the plants’ cellular resilience to heat stress. Chlorophylls, essential pigments for photosynthesis, serve as critical indicators of plant vitality and photosynthetic capacity (Mihaljević et al., 2020). BR and SA treatments significantly enhanced the SPAD value of leaves under high-temperature conditions and reduced relative electrolyte conductivity (REC) values, thereby protecting cell integrity and morphology.

### Hormone treatments alleviate the decline in fruit quality

In this experiment, high temperatures during the flowering period significantly reduced the fruit set rate of ‘Kyoho’ grapevines. However, treatments with BR and SA improved the visual quality of the grapes compared to the heat stress (HT) group, with increases in fruit set rate of 22.67% and 13%, respectively (**Figure 7**). Heat stress negatively impacted fruit weight over time, primarily due to shortened berry development and accelerated fruit ripening. This hindered the accumulation of assimilates in the berries, as previously observed (Hewitt et al., 2023). Additionally, water evaporation from berries was faster under heat stress, leading to reduced fruit water content (Santesteban et al., 2011). Heat stress also inhibited anthocyanin accumulation, a key factor in fruit coloration. BR treatment mitigated this effect by enhancing leaf photosynthesis and promoting anthocyanin synthesis, thereby improving fruit coloration (Lv et al., 2022). In this study, the appearance quality of grapes subjected to heat stress from veraison to maturity was significantly lower than that of the control (CK) group. Specifically, fruit weight decreased by 27.84%, longitudinal and transverse berry diameters were reduced by 14.55% and 9.7%, respectively, and total anthocyanin content declined by 53.17%. These findings align with previous studies. Sugar and acid contents are essential indicators of grape quality. During fruit ripening, heat stress increases fruit potassium concentration, which raises pH levels and ultimately reduces total acidity (Neethling et al., 2012; Bernardo et al., 2018). In this study, heat stress slowed the synthesis rate of soluble solids (TSS) while accelerating the degradation of total acidity (TA). Reduced photosynthetic efficiency in leaves under heat stress led to an insufficient supply of organic nutrients to meet the plant’s demand. Consequently, TSS synthesis was insufficient, resulting in a reduction of monosaccharide components, such as glucose and fructose, in the berries. These findings indicate that high temperatures significantly impair the synthesis of fructose and glucose, contributing to a decrease in total soluble sugar content in the fruit. Exogenous BR application showed a marked improvement in the flavor profile of the fruit. An appropriate concentration of BR significantly enhanced fruit sugar content and promoted coloration (Ghorbani et al., 2017). Similarly, SA treatment positively affected fruit quality by increasing the fruit set rate, improving fruit weight and size, and enhancing anthocyanin content to levels comparable to those observed with BR treatment.

The application of BR has a more pronounced effect than SA in enhancing the Pnmax, maintaining light and CO_2_ saturation points, and improving the photochemical efficiency of photosystem II (PSII). This enhanced performance indicates that BR may more effectively mitigate heat-induced inhibition of photosynthesis by promoting stomatal opening and enhancing CO_2_ assimilation (Li et al., 2024). In contrast, SA primarily activates stress-responsive defense pathways, such as antioxidant and pathogen resistance pathways, exhibits a comparatively weaker systemic adaptation to sustained heat stress (Preet et al., 2023). These mechanistic differences are most evident during heat-sensitive stages, such as flowering and berry expansion, ultimately influencing yield and fruit quality.

## Conclusion and further research

The results of this study demonstrate that exogenous application of hormones, specifically brassinosteroids (BR) and salicylic acid (SA), as a nutrient solution to the roots, significantly alleviated the adverse effects of heat stress on ‘Kyoho’ grapevines. Both treatments effectively enhanced photosynthetic performance during the flowering, young berry swelling, and veraison stages by increasing the net photosynthetic rate (Pn), maintaining light and CO_2_ saturation points, and improving PSII photochemical efficiency. These effects mitigated PSII photoinhibition caused by high temperatures, with BR showing the most pronounced efficacy. Additionally, BR and SA treatments reduced malondialdehyde (MDA) content in the leaves, alleviating membrane lipid peroxidation caused by heat stress. Both treatments also increased soluble protein (SP) content, offering further protection to leaf tissues. BR treatment was particularly effective in improving the fruit set rate, promoting berry expansion, and enhancing the accumulation of key quality components such as fructose, glucose, and anthocyanins in the fruit skin. While SA exhibited similar benefits, its effects were generally less pronounced compared to BR. These findings highlight the potential of BR and SA as effective treatments for enhancing grapevine resilience and fruit quality under high-temperature conditions. Applying hormones through root irrigation offers a practical approach for vineyard management. provides valuable insights into the effects of hormonal treatments across key growth stages, including flowering, berry swelling, and veraison. However, the experiment was conducted in a controlled greenhouse environment, which may not fully reflect field conditions, including fluctuating temperatures, varying light exposure, and diverse soil types. Additionally, the potential synergistic effects of combining BR and SA treatments were not explored. Future research should focus on examining the combined application of BR and SA to understand their potential synergistic effects on grapevine resilience to heat stress. Exploring the interaction between these hormones could provide additional insights into their mechanisms of action and reveal more effective strategies for managing heat stress in vineyards.

## Data Availability

The original contributions presented in the study are included in the article/**Supplementary Material**. Further inquiries can be directed to the corresponding author.
