# Supplementary material for: Root-applied brassinosteroid and salicylic acid enhance thermotolerance and fruit quality in heat-stressed ‘Kyoho’ grapevines
Source: Front Plant Sci. 2025 Apr 3;16:1563270. doi: 10.3389/fpls.2025.1563270 (PMC12003391; doi:10.3389/fpls.2025.1563270)
Supplement: Supplementary file 1 [file Table1.docx]

Supplementary Material

# Supplementary Table

Table 1. Pretest concentration gradient

| **Concentration** | EBR (ppm) | SA (μmol·L⁻¹) |
| --- | --- | --- |
| Low concentration | 200 | 100 |
| Intermediate concentration | 400 | 150 |
| High concentration | 800 | 200 |
